# Factors associated with chronic obstructive pulmonary disease exacerbation, based on big data analysis

**DOI:** 10.1038/s41598-019-43167-w

**Published:** 2019-04-30

**Authors:** Jongmin Lee, Hyun Myung Jung, Sook Kyung Kim, Kwang Ha Yoo, Ki-Suck Jung, Sang Haak Lee, Chin Kook Rhee

**Affiliations:** 1 Division of Pulmonary, Allergy and Critical Care Medicine, Department of Internal Medicine, Seoul St Mary’s Hospital, College of Medicine, The Catholic University of Korea, Seoul, Republic of Korea; 2 CC&I Research, Seoul, Republic of Korea; 3 Division of Pulmonary, Allergy, and Critical Care, Medicine, Department of Internal Medicine, Konkuk University School of Medicine, Seoul, Republic of Korea; 4 Division of Pulmonary, Allergy, and Critical Care Medicine, Department of Internal Medicine, Hallym University Sacred Heart Hospital, Hallym University College of Medicine, Anyang, Republic of Korea; 5 Division of Pulmonary, Allergy, and Critical Care Medicine, Department of Internal Medicine, St Paul’s Hospital, College of Medicine, The Catholic University of Korea, Seoul, Republic of Korea

**Keywords:** Chronic obstructive pulmonary disease, Chronic obstructive pulmonary disease, Outcomes research, Outcomes research

## Abstract

Preventing exacerbation in chronic obstructive pulmonary disease (COPD) patients is crucial, but requires identification of the exacerbating factors. To date, no integrated analysis of patient-derived and external factors has been reported. To identify factors associated with COPD exacerbation, we collected data, including smoking status, lung function, and COPD assessment test scores, from 594 COPD patients in the Korean COPD subgroup study (KOCOSS), and merged these data with patients’ Korean Health Insurance Review and Assessment Service data for 2007–2012. We also collected primary weather variables, including levels of particulate matter <10 microns in diameter, daily minimum ambient temperature, as well as respiratory virus activities, and the logs of web queries on COPD-related issues. We then assessed the associations between these patient-derived and external factors and COPD exacerbations. Univariate analysis showed that patient factors, air pollution, various types of viruses, temperature, and the number of COPD-related web queries were associated with COPD exacerbation. Multivariate analysis revealed that the number of exacerbations in the preceding year, female sex, COPD grade, and influenza virus detection rate, and lowest temperature showed significant association with exacerbation. Our findings may help COPD patients predict when exacerbations are likely, and provide intervention as early as possible.

## Introduction

Chronic obstructive pulmonary disease (COPD) characteristically involves an airflow limitation that is not fully reversible. Its worldwide prevalence is increasing, and the Global Burden of Disease Study has estimated that COPD will be the fourth leading cause of death by 2030^1^. Although pharmacotherapies for COPD have improved, many patients still experience exacerbations of COPD, during which respiratory symptoms worsen acutely, and which determine disease-associated morbidity, mortality, resource burden, and healthcare costs^2^. After exacerbations, the patients’ symptomatic and pulmonary function recovery takes several weeks, and their quality of life may be seriously degraded. In a large study of commercially insured COPD patients, the total medical and pharmaceutical costs per patient admitted to an emergency department or as a hospital inpatient was approximately $2,000–$40,000^3^. Hence, it is extremely important to prevent acute exacerbation in COPD patients, but this requires identification of the factors associated with exacerbation.

To date, most research on COPD-exacerbating factors has relied on cohort data. Moreover, most studies have focused on factors inherent to the COPD patients themselves, rather than external factors^4–6^. Although external factors, such as air pollution and viral infection, are known to contribute to COPD exacerbation^7–9^, few studies have analysed the factors associated with COPD based on cohort as well as external data.

Korea has a compulsory universal health insurance system that includes medical reimbursement records for the entire Korean population. All hospitals, clinics, public health centres, and pharmacies are registered with the Korean National Health Insurance. The Korean Health Insurance Review and Assessment Service (HIRA) database contains detailed information about diagnosis, health care use, and medication, and is a reliable source for nationwide epidemiological evaluations^10^. Merging these claims data with cohort data provides a powerful research resource. Cohort data contain very detailed and accurate information regarding COPD, such as lung function, quality of life, and smoking status, which are usually lacking in claims data. Furthermore, in Korea, big data on factors that can potentially influence acute exacerbations, such as air pollution, social network services, weather data, and respiratory virus detection rates, are available.

However, no previous study has collected and integrated all these potential factors in COPD research. We therefore analysed such combined big data in order to identify factors associated with acute exacerbation of COPD.

## Results

### Baseline patient characteristics

Supplementary Table S1 shows the baseline characteristics of the COPD patients who were included in this study. Their average age was 65.0 ± 7.5 years, and most were male (n = 538, 90.6%). Almost all patients (98.8%) were current or former smokers. The post-bronchodilator FEV1 was 55.9 ± 17.2% predicted, and the FEV1/FVC ratio was 48.3 ± 12.0. The mean CAT score was 15.8 ± 7.4 and the CAT score was ≥10 in 79.0% of the patients. Of the total study population, the percentages of patients in GOLD stages I, II, III, and IV were 6.7 (n = 38), 53.9 (n = 305), 33.9 (n = 192), and 5.5 (n = 31).

### Patient characteristics associated with COPD acute exacerbation during a 5-year follow-up period

Univariate analysis was used to examine the influence of patient characteristics on COPD acute exacerbations during a 5-year follow-up period, and the results are shown in Supplementary Table S2. Old age, female sex, smoking status, high CAT score, low FEV1, higher grade of COPD, number of exacerbations during the previous year (2007), and number of visits to the ER during the previous year (2007) were all associated with COPD acute exacerbation.

### Meteorological factors associated with COPD acute exacerbation during a 5-year follow-up period

**Figure 1:**
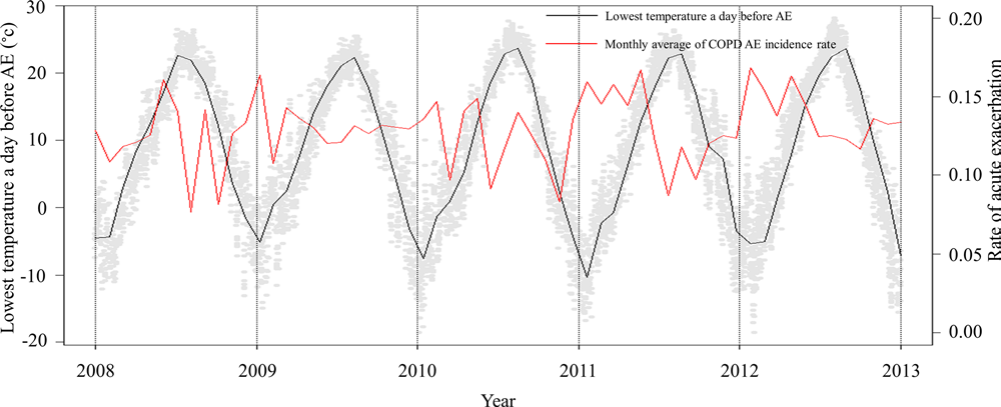
Relationship between the lowest temperature and chronic obstructive pulmonary disease (COPD) acute exacerbation.

Table 1 shows the influence of environmental factors on COPD acute exacerbation in terms of univariate analysis. Humidity, variation of diurnal temperature, lowest temperature, and cumulative amount of rainfall during the 7 days before an acute exacerbation were associated with acute exacerbations. Figure 1 shows the effect of the lowest temperature 1 day prior to a COPD acute exacerbation. The lowest temperature was also correlated with PM10 and the virus detection rate (as detailed in Supplementary Table S3).

**Table 1:** Univariate analysis of environmental factors to identify factors predicting chronic obstructive pulmonary disease (COPD) acute exacerbations.

	OR (95% CI)	*P*-value
Average of humidity	0.9967 (0.9930–1.0004)	0.0137
Diurnal temperature variation	1.0133 (0.9931–1.0339)	0.0472
Hours of daylight	1.0064 (0.9954–1.0176)	0.2362
Lowest temperature a day before AE	0.9945 (0.9894–0.9997)	0.0060
Lowest temperature at AE	0.9950 (0.9899–1.0001)	0.0108
Cumulative lowest temperature of 3 days before AE	0.9982 (0.9964–0.9999)	0.0068
Cumulative lowest temperature of 5 days before AE	0.9989 (0.9978–0.9999)	0.0050
Cumulative lowest temperature of 7 days before AE	0.9991 (0.9984–0.9999)	0.0030
Maximum wind velocity	1.0198 (0.9704–1.0718)	0.1470
Cumulative rainfall of 3 days before AE	0.9994 (0.9981–1.0006)	0.3470
Cumulative rainfall of 5 days before AE	0.9992 (0.9982–1.0002)	0.1108
Cumulative rainfall of 7 days before AE	0.9992 (0.9985–1.0000)	0.0389

OR, Odds ratio; AE, acute exacerbation.

### Air pollution factors associated with COPD acute exacerbation during a 5-year follow-up period

**Figure 2:**
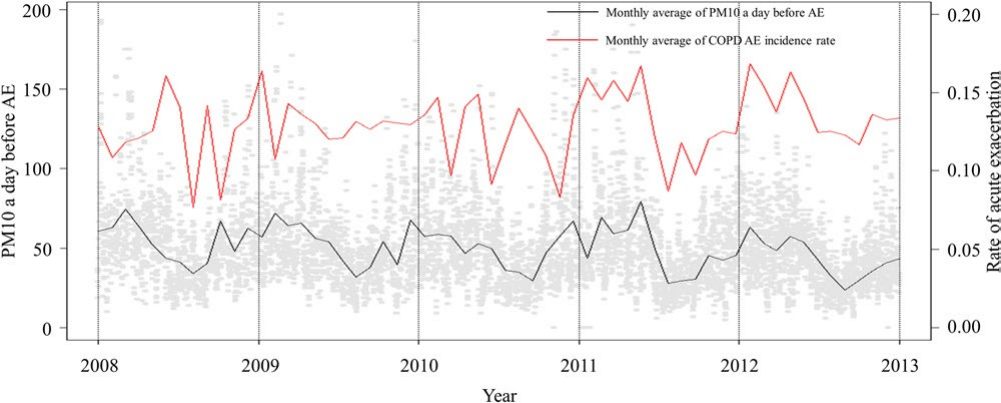
Correlation between PM10 and chronic obstructive pulmonary disease (COPD) acute exacerbation.

The results of univariate analyses, showing the relationship between air pollution and COPD exacerbation, are shown in Table 2. PM10 levels 1 day before acute exacerbation was associated with acute exacerbation. The monthly mean incidence rate of COPD acute exacerbation showed a similar tendency with PM10 1 day prior to the acute exacerbation (Fig. 2).

**Table 2:** Univariate analysis of air pollution factors for the prediction of chronic obstructive pulmonary disease (COPD) acute exacerbations.

	OR (95% CI)	*P*-value
PM10 a day before AE	1.0017 (1.0000–1.0034)	0.0260
Cumulative PM10 of 3 days before AE	1.0006 (0.9999–1.0013)	0.0522
Cumulative PM10 of 5 days before AE	1.0003 (0.9998–1.0008)	0.1211
Cumulative PM10 of 7 days before AE	1.0002 (0.9998–1.0006)	0.1622

OR, odds ratio; PM, particulate matter; AE, acute exacerbation.

### Web query data associated with COPD acute exacerbation during a 5-year follow-up period

The results of GEE model analysis of the relationship between web search data and COPD exacerbations are shown in Supplementary Table S4. In univariate analysis, the number of logs of online web search queries about COPD, containing the words “flu”, “dyspnea”, “asthma”, “COPD”, “acute exacerbation”, “emphysema” and “chronic bronchitis” at 1 and 2 weeks prior to exacerbation correlated positively with COPD exacerbation.

### Viral factors associated with COPD acute exacerbation during a 5-year follow-up period

**Figure 3:**
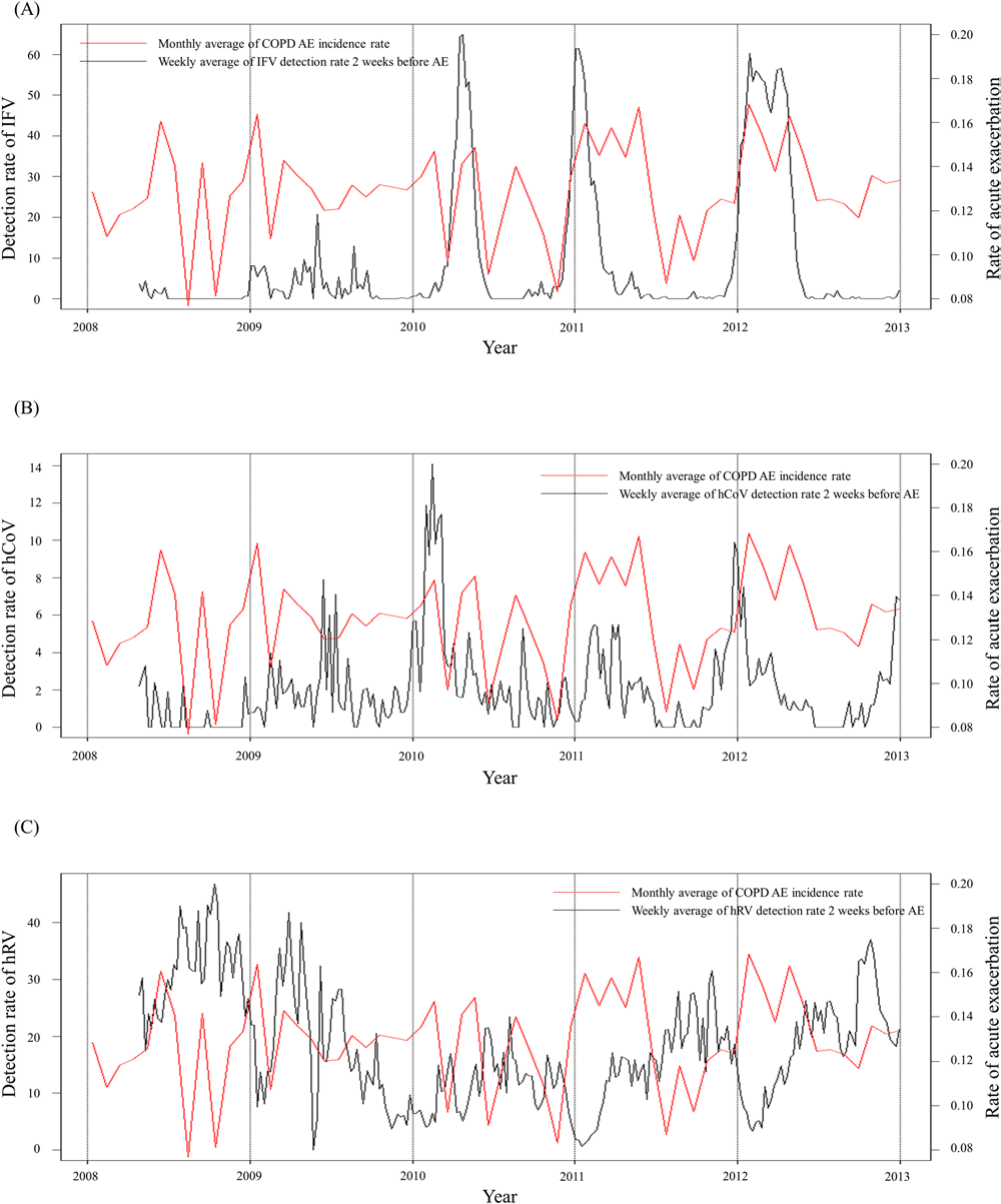
Relationship between the detection rate of virus and chronic obstructive pulmonary disease (COPD) acute exacerbation. (**A**) Relationship between influenza virus (IFV) and COPD acute exacerbation. (**B**) Relationship between human corona virus (hCoV) and COPD acute exacerbation. (**C**) Relationship between human rhinovirus (hRV) and COPD acute exacerbation.

Univariate analysis results indicating the relationship between viral factors and COPD acute exacerbation are shown in Table 3. The detection rate of IFV, hCoV, hRV, and COPD acute exacerbation correlated positively. Figure 3 shows the relationship between the detection rate of viruses and COPD acute exacerbation.

**Table 3:** Univariate analysis of viral factors for the prediction of chronic obstructive pulmonary disease (COPD) acute exacerbations.

	OR (95% CI)	*P*-value
Detection rate of ADV 2 weeks before AE	0.9940 (0.9817–1.0064)	0.1351
Detection rate of IFV 2 weeks before AE	1.0058 (1.0027–1.0089)	<0.0001
Detection rate of PIV 2 weeks before AE	0.9968 (0.9847–1.0091)	0.3923
Detection rate of RSV 2 weeks before AE	0.9963 (0.9867–1.0060)	0.3079
Detection rate of hBoV 2 weeks before AE	0.9864 (0.9641–1.0091)	0.1299
Detection rate of hCoV 2 weeks before AE	1.0215 (0.9991–1.0444)	0.0187
Detection rate of hEV 2 weeks before AE	0.9895 (0.9579–1.0220)	0.2925
Detection rate of hRV 2 weeks before AE	0.9928 (0.9872–0.9983)	0.0010
Cumulative detection rate of ADV 2–5 weeks before AE	0.9991 (0.9956–1.0025)	0.3863
Cumulative detection rate of IFV 2–5 weeks before AE	1.0015 (1.0007–1.0023)	<0.0001
Cumulative detection rate of PIV 2–5 weeks before AE	0.9989 (0.9956–1.0023)	0.2799
Cumulative detection rate of RSV 2–5 weeks before AE	0.9997 (0.9971–1.0024)	0.7916
Cumulative detection rate of hBoV 2–5 weeks before AE	0.9951 (0.9885–1.0017)	0.0541
Cumulative detection rate of hCoV 2–5 weeks before AE	1.0072 (1.0009–1.0135)	0.0050
Cumulative detection rate of hEV 2–5 weeks before AE	0.9965 (0.9874–1.0057)	0.2250
Cumulative detection rate of hRV 2–5 weeks before AE	0.9984 (0.9969–0.9999)	0.0057

OR, odds ratio; ADV, influenza adenovirus; IFV, influenza virus; PIV, parainfluenza virus; RSV, respiratory syncytial virus; hBoV, human bocavirus; hCoV, human coronavirus; hEV, human enterovirus; hRV, human rhinovirus.

Table 4 shows multivariate associations between potential predictor variables and exacerbations. Female sex, number of exacerbations in the baseline year, COPD grade, and detection rate of IFV 2 weeks before acute exacerbation were significantly positively correlated with COPD acute exacerbation. Multivariate analysis revealed that the number of exacerbations in the preceding year, female sex, COPD grade, and IFV detection rate, and lowest temperature showed significant association with exacerbation

**Table 4:** Factors significantly associated with chronic obstructive pulmonary disease (COPD) acute exacerbations, estimated by a generalized estimation equation model.

	OR (95% CI)	*P*-value
Female sex	1.5596 (1.1742–2.0715)	0.0003
Number of exacerbations during a previous year (2007)	1.2745 (1.2095–1.3430)	<0.0001
COPD grade	1.5693 (1.1727–2.1001)	<0.0001
Detection rate of IFV 2 weeks before AE	1.0075 (1.0017–1.0133)	0.0007
Cumulative lowest temperature of 5 days before AE	0.9572 (0.9219–0.9938)	0.0170
Lowest temperature a day before AE	0.9587 (0.9196–0.9994)	0.0474

OR, odds ratio; IFV, influenza virus; AE, acute exacerbation.

Factors are adjusted by age, FEV1 (%), COPD assessment (CAT) score, number of hospital admissions during the previous year, number of ER visits during the previous year, prescription of COPD medication, ICD-10 code for comorbidities, meteorological data, air pollution data, virus data, and web search data.

## Discussion

**Figure 4:**
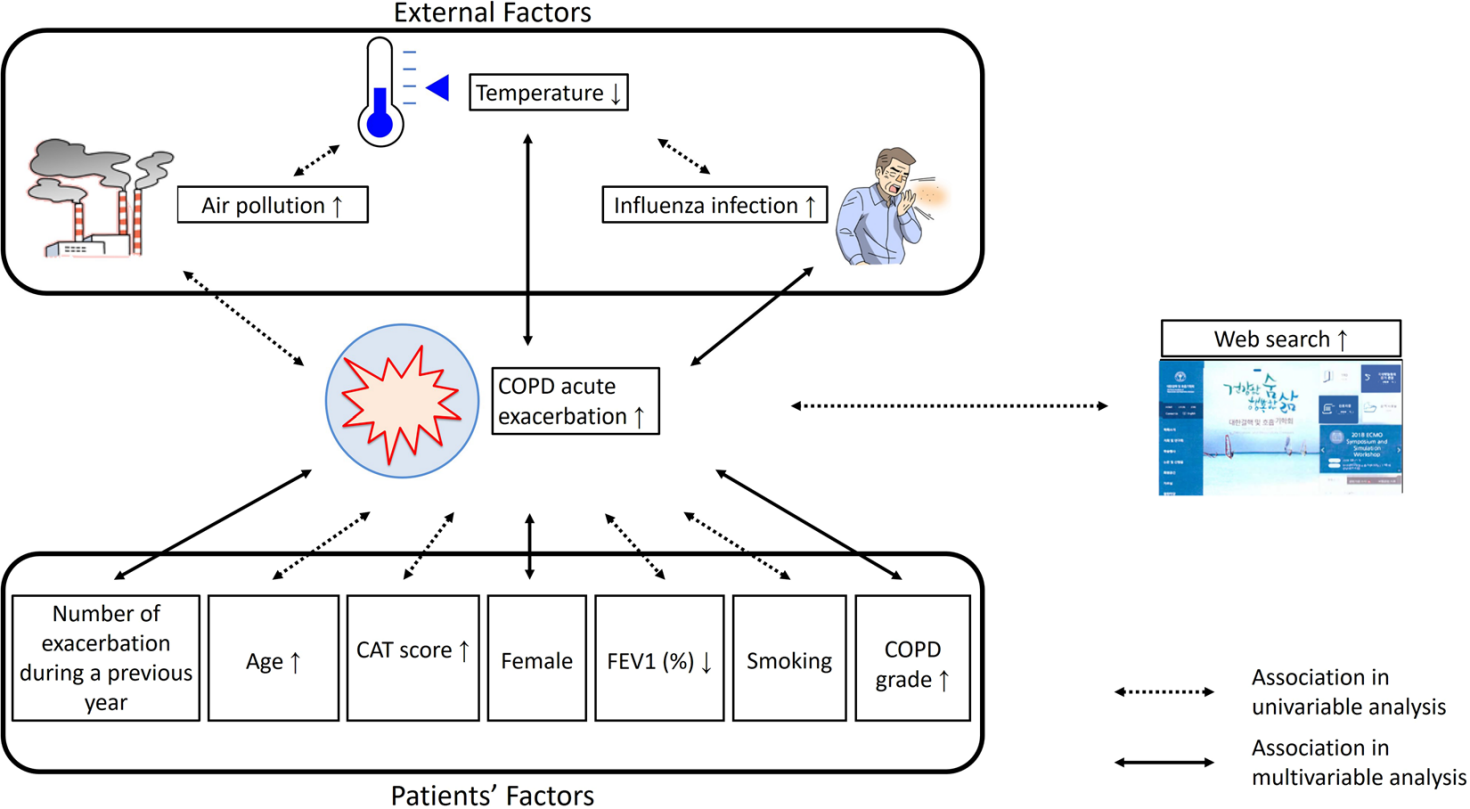
Summary of results of this study: association between external patient-derived factors and chronic obstructive pulmonary disease (COPD) acute exacerbation. Reprinted with permission of the The Korea Academy of Tuberculosis and Respiratory Diseases. Copyright © The Korea Academy of Tuberculosis and Respiratory Diseases. Author (s)/Year/Title/ Pages.

We merged clinical data from cohort and 6 years’ follow-up claims data with external factors, such as air pollution and viral infection rates, to identify factors that were associated with acute COPD exacerbation. The possible factors associated with exacerbation were female sex, a history of frequent exacerbations in the previous year, a higher COPD grade, the IFV detection rate in the period before an acute exacerbation, and a low lowest temperature before an acute exacerbation (Fig. 4).

Many other predictive studies have focused only on factors inherent to COPD patients^4–6,11^, or only on external factors, such as environmental or viral factors^8,9,12–15^. In the present study, we integrated factors related to the COPD patients with external potentially contributing factors.

Previous studies on predictors of COPD exacerbation have commonly identified airway obstruction (FEV1% predicted, or FEV1, or GOLD stage), previous exacerbations, age, and smoking as the causes of COPD exacerbation^16^. Similar to previous studies, a higher grade of COPD and the number of exacerbations were identified as significant predictors; older age and smoking status were significant in the present univariate, but not multivariate analyses.

A lower lowest temperature and the viral detection rate were related with COPD exacerbation. COPD exacerbations were more common in the winter months, with colder temperature, when respiratory viral infections are more prevalent in the community^9,17^. A two-fold increase in the COPD exacerbation rate in winter has previously been reported^18,19^, and a previous nationwide study has shown that cold temperature increased COPD exacerbation^14^, in agreement with the present findings.

Viral infection is a major cause of COPD exacerbations; the respiratory viruses most frequently involved in COPD exacerbations are rhinoviruses, IFVs, coronaviruses, and respiratory syncytial virus^9,20–22^. Papi *et al*. have reported that viral infection played a role in 48% of COPD exacerbations; based on polymerase chain reaction analyses, rhinovirus infections were the most common, followed by IFVs, accounting for 23% of virus-associated exacerbations^22^. The present univariate analysis implicated various viruses in COPD exacerbation, as in previous studies. However, in multivariate analysis, the correlation between viruses other than IFV and COPD exacerbation was not statistically significant, perhaps due to the confounding effect of temperature, which is a very powerful factor in COPD exacerbation.

In this study, PM10 was identified as a significant predictor in univariate analysis, and the monthly mean trend of PM10 showed a similar tendency as that of the COPD exacerbation incidence rate. Due to the rapid urbanization of the world population, air pollution has become a major health problem. Outdoor air pollution also seems to be an important environmental trigger for acute exacerbation of COPD^23^. A few studies have reported the relationship between air pollution, such as particulate matters (PM10, PM2.5) and harmful gases (NO_2_, SO_2_, and O_3_), and COPD exacerbation^7,24–26^. The deposition of PM in the respiratory tract depends on the size of the particles, and its insufficient clearance may cause a chronic, low-grade inflammatory response, which is known to cause COPD exacerbations^27^. Currently, there is insufficient evidence that air pollution is a causative factor of COPD, further studies on this topic are therefore recommended. Although multivariate analysis showed that the correlation between PM10 and COPD exacerbation was not statistically significant in this study, this discrepancy could also be attributed to the powerful factor of temperature.

Lower temperature, a higher virus detection rate, and a higher concentration of PM10 were significantly correlated with COPD exacerbation in the present study. This result suggested that cold weather eventually increases viral infection and air pollution. Multivariate analysis showed that COPD exacerbations are not related to viruses other than influenza, and air pollution, but this is assumed to have been due to the temperature factor, which affects viral infection and air pollution, rather than due to their actual irrelevance.

Our study demonstrated that the number of exacerbations during the previous year is a significant predictive factor, which was in accord with the findings of previous studies. Make *et al*. reported that a history of prior exacerbations and more exacerbations during a previous year were strong predictors for future exacerbations^28^. Müllerová *et al*. also reported that patients with only one prior moderate exacerbation were at increased risk of future exacerbation events^29^. A large observational cohort study also demonstrated that the single best predictor of exacerbations was a history of previous exacerbations^30^. The findings of these studies were consistent with those reported in the present study.

Our study had several strengths. First, this study analysed both patient factors and external factors. Furthermore, we merged our cohort data with claims data. Cohort data contained detailed and accurate information regarding COPD, such as lung function and CAT score, while claims data included medical reimbursement records for the entire Korean population, and allowed us to obtain the exact date of COPD exacerbation. Using this information, we could precisely match COPD exacerbations with external factors, which included information on the weather, air pollution, and viruses. Moreover, no previous study had analysed COPD patients using nationwide big data or long-term follow-up (5 years) data.

The study had some limitations. First, COPD exacerbation might be under or overestimated in this study, because the exacerbation data analysed were based on claims data, rather than being acquired via an exacerbation diary or patient interview. Second, since the concentration of PM and the detection rate of viruses vary according to region, consideration should be given to the local distribution of patients. We had, in fact, attempted to adjust for the local distributions of patients based on claims data. It is possible to identify the location of the hospital from the HIRA data; however, this adjustment is not perfect, since the patient may visit a hospital far from home. A further study that considers the regional distribution is therefore required. Third, some of the baseline data were adopted from the cohort data, and there was a time difference between cohort data and HIRA data. Thus, CAT, PFT, BMI, smoking status were actually measured after the follow-up period. Also, difference of data measurement period (e.g., daily mean PM10 and weekly viral infection status) should be considered as the limitation of this study. Finally, we did not weight on any variables in multivariate analysis. The methodology we developed in this study is the first attempt in the world, and there has been no reference how to weight in equation. Further study with large number of patients will be needed.

In conclusion, in this study, we merged factors inherent to COPD patients and external factors, such as environmental, viral, and web search data, to identify factors associated with acute exacerbation of COPD. Univariate analysis showed that patient factors, air pollution, various types of viruses, temperature, and the number of web queries about COPD were associated with COPD exacerbation. We demonstrated that the number of exacerbations in the preceding year, being female, a high grade of COPD, the IFV detection rate, and a low lowest temperature were significantly associated with future exacerbation events, according to multivariate analysis. Since exacerbations can negatively impact health status and disease progression^31,32^, these findings may help to identify the COPD patients who are at risk of exacerbation and to provide intervention as early as possible.

## Methods

### Study design and data source

We investigated data from 594 COPD patients who were enrolled in the Korean COPD subgroup study (KOCOSS) cohort between December 2011 and March 2014. Patients were eligible if they had been diagnosed with COPD by a pulmonologist, were aged ≥40 years, had post-bronchodilator FEV1/FVC < 0.7, and if respiratory symptoms, such as cough, sputum, or dyspnoea, were present. Detailed information regarding KOCOSS cohort is described in a previous publication^33^.

We obtained four data items from KOCOSS cohort, i.e., smoking status, lung function, body mass index (BMI), and COPD assessment test (CAT) scores. We then merged these items with their HIRA data from between 2007 and 2012; the latter data included details on comorbidity, medication, and health care utilization. Moderate exacerbation was defined as when COPD patients visited outpatient clinics with an ICD-10 code of COPD (J43.x−44.x, except J430, as the primary or within the fifth secondary diagnosis) and systemic steroid medication with or without antibiotics were prescribed. Severe exacerbation was defined as when COPD patients visited the emergency room or were admitted to hospital with an ICD-10 code of COPD and were prescribed steroid medication with or without antibiotics. A high COPD grade was arbitrarily defined as patients who were (1) tertiary hospital care patients meeting the above definition of COPD, and (2) regularly used triple inhaler therapy (inhaled corticosteroids [ICS] + long acting beta-2 agonists [LABA] + long acting muscarinic antagonists [LAMA]) or used of systemic steroid therapy at least twice per year with COPD inhaler therapy (LAMA or ICS + LABA)^34^. The HIRA 2007–2012 data were used to analyse utilization of COPD medication, comorbidity, and hospital admission. The 2007 data were used to identify the history of COPD exacerbation over the past year, and the 2008–2012 data were used to analyse the relationship between COPD exacerbation and putative predictive factors.

We also collected primary weather variables, including levels of particulate matter <10 microns in diameter (PM10, µg/m^3^), daily minimum ambient temperature (°C), and daily precipitation for 2007–2012. Daily local meteorological weather data, measured at the local weather stations, were provided by the Korean Ministry of the Environment. All the information amassed was matched to the patients’ addresses. Matching was performed at province level. The area of the respective provinces (n = 16) ranged from 501 to 19,031 km^2^ (median: 6,628 km^2^, interquartile range: 827–10,362 km^2^).

The Korean National Institute of Health (KNIH) monitors the trends of virus activity on a nation level. KNIH has surveyed respiratory viral infection status since 2000 and provided information to the public on a weekly basis (detection rate, %). Data on the activities of influenza adenovirus, parainfluenza virus, respiratory syncytial virus, influenza virus (IFV), human coronavirus (hCoV), human rhinovirus (hRV), human bocavirus, and human enterovirus were collected from the KNIH database for the 2007–2012 period.

Additionally, we tracked the tendencies of online web search queries (normalized search volume index, 0–100), containing the key terms “flu”, “dyspnea”, “asthma”, “COPD”, “acute exacerbation”, “emphysema,” and “chronic bronchitis” on Naver (www.naver.com; the biggest search engine site in Korea), for the period 2007 to 2012.

### Statistical analysis

Descriptive statistics were used to characterize the study population (mean and standard deviation [SD], and percent). Independent *t*-tests were used to compare continuous variables and chi-square tests were used for categorical variable comparisons. Because all longitudinal data related to these patients are measured repeatedly, it violates the independency assumption of general statistical techniques. Therefore, the occurrence of acute exacerbation was investigated using generalized estimating equations (GEEs)^35^. Univariate analysis was performed by GEE between COPD exacerbation and single factor one by one. Then, multivariable analysis was performed with all factors included. Factors included in multivariable analysis were time variables, sex, smoking history, CAT score, FEV1(%), prescription of COPD medication, ICD-10 code for comorbidities, number of hospital admissions during the previous year (2007), number of ER visits during the previous year (2007), the history of acute exacerbations during the previous year (2007), grade of COPD, activities of viruses, cumulative exposures to PM10, meteorological factors, and online web search query logs. Subsequently, backward elimination of significant variables was performed. Pearson’s correlation coefficient and Spearman’s correlation coefficient were used to assess the relationships between lowest temperature and other variables. All analyses were performed with SAS software, version 9.4 (SAS Institute, Cary, NC).

## Supplementary information

Supplementary Material
