# Supplementary material for: Factors associated with chronic obstructive pulmonary disease exacerbation, based on big data analysis
Source: Sci Rep. 2019 Apr 30;9:6679. doi: 10.1038/s41598-019-43167-w (PMC6491439; doi:10.1038/s41598-019-43167-w)
Supplement: Supplementary file 1 — Supplementary Material [file 41598_2019_43167_MOESM1_ESM.docx]

**Supplementary Material**

***Factors associated with COPD exacerbation, based on big data analysis***

**Appendix: Additional Tables**

**Table S1** Baseline characteristics of the patients

**Table S2** Univariate analysis of patient characteristics to identify factors predicting COPD acute exacerbations

**Table S3** Correlation of lowest temperature with PM10 and detection rate of viruses

**Table S4** Univariate analysis of amount of web search queries about COPD for the prediction of COPD acute exacerbations

Table S1. Baseline characteristics of the patients

| **Variables** | **No. of subjects** | **No. (%) or observation** |
| --- | --- | --- |
| Age, year, mean ± SD | 594 | 65.0 ± 7.5 |
| Male, No. (%) | 594 | 538 (90.6) |
| BMI, kg/m2, mean ± SD | 574 | 22.8 ± 3.4 |
| Smoking, pack/yr, mean ± SD | 505 |  |
| Current smoker, % |  | 149 (29.5) |
| Former smoker, % |  | 350 (69.3) |
| Non-smoker, % |  | 6 (1.2) |
| Lung function |  |  |
| FEV1/FVC, %, mean ± SD | 567 | 48.3 ± 12.0 |
| Post-bronchodilator FEV1, %, mean ± SD | 566 | 55.9 ± 17.2 |
| Post-bronchodilator FEV1, L, mean ± SD | 568 | 1.5 ± 0.5 |
| FVC, L, mean ± SD | 587 | 3.0 ± 0.8 |
| TLC, L, mean ± SD | 379 | 110.7 ± 24.9 |
| Symptom scores |  |  |
| CAT, mean ± SD | 544 | 15.8 ± 7.4 |
| CAT score < 10 |  | 114 (21.0) |
| CAT score ≥ 10 |  | 430 (79.0) |
| GOLD stage (I to IV) | 566 |  |
| I |  | 38 (6.7) |
| II |  | 305 (53.9) |
| III |  | 192 (33.9) |
| IV |  | 31 (5.5) |

BMI, body mass index; CAT, COPD assessment test.

Table S2. Univariate analysis of patient characteristics to identify factors predicting COPD acute exacerbations.

|  | **OR (95% CI)** | ***P*-value** |
| --- | --- | --- |
| Age | 1.0108 (0.9857 – 1.0366) | 0.0003 |
| Female sex | 1.7104 (0.9366 – 3.1234) | <0.0001 |
| Smoking status |  |  |
| Never smoker | 1 |  |
| Ex-smoker | 4.8036 (3.1379 – 7.3534) | 0.0002 |
| Current smoker | 4.6574 (2.6306 – 8.2458) | 0.0002 |
| CAT score | 1.0333 (1.0091 – 1.0580) | <0.0001 |
| FEV1 | 0.9833 (0.9715 – 0.9952) | <0.0001 |
| High COPD grade | 1.1988 (0.8876 – 1.6192) | <0.0001 |
| Number of exacerbations during a previous year (2007) | 1.1801 (1.1123 – 1.2519) | <0.0001 |
| Number of visiting ER during a previous year (2007) | 1.6760 (1.4497 – 1.9377) | <0.0001 |

OR, Odds ratio; CAT, COPD assessment test.

Table S3. Correlation of lowest temperature with PM10 and detection rate of viruses

|  | **Lowest temperature** | |
| --- | --- | --- |
|  | **r** | ***P*-value** |
| PM10 | -0.23644 | <0.0001 |
| Detection rate of ADV | 0.15666 | <0.0001 |
| Detection rate of PIV | 0.42751 | <0.0001 |
| Detection rate of RSV | -0.39614 | <0.0001 |
| Detection rate of IFV | -0.45074 | <0.0001 |
| Detection rate of hCoV | -0.45090 | <0.0001 |
| Detection rate of hRV | 0.47014 | <0.0001 |
| Detection rate of hBoV | 0.37075 | <0.0001 |
| Detection rate of hEV | 0.13113 | <0.0001 |

r = Pearson correlation coefficient for PM10, Spearman correlation coefficient for detection rate of viruses; ADV, influenza adenovirus; PIV, parainfluenza virus; RSV, respiratory syncytial virus; IFV, influenza virus; hCoV, human coronavirus; hRV, human rhinovirus; hBoV, human bocavirus; hEV, human enterovirus.

Table S4. Univariate analysis of amount of web search queries about COPD for the prediction of COPD acute exacerbations

|  | **OR (95% CI)** | ***P*-value** |
| --- | --- | --- |
| Amount of web search quarries about COPD a week before AE | 1.0015 (0.9993 – 1.0037) | 0.0099 |
| Amount of web search quarries about COPD during 2 weeks before AE | 1.0007 (0.9995 – 1.0019) | 0.0201 |
| Amount of web search quarries about COPD during 4 weeks before AE | 1.0003 (0.9997 – 1.0009) | 0.0699 |

OR, odds ratio; AE, acute exacerbation.
